# Research on Time-Driven Activity-Based Management System of Public Hospitals

**DOI:** 10.3389/fpubh.2021.763829

**Published:** 2022-01-27

**Authors:** Qiwen Jiang, Xueyuan Zhu, Lianghua Chen, Ziyuan Zhao, Yilong Chen

**Affiliations:** ^1^School of Economic Management, Southeast University, Nanjing, China; ^2^School of Economics, Finance and Accounting, Coventry University, Coventry, United Kingdom

**Keywords:** public hospitals, time-driven activity-based costing, cost management, precision, working efficiency

## Abstract

**Objective:**

To provide references for effective implementing cost management for public hospitals through establishing time-driven activity-based management (TDABM) system. The TDABM system was established from hospital cost accounting, budget, control, and performance.

**Results:**

The established TDABM system could improve the precision of hospital cost accounting, improve medical staff's working efficiency, realize the whole process of cost management, and enhance the competitiveness of the hospital.

**Conclusion:**

The activity of implementing TDABM in public hospitals had practical significance.

## Introduction

It is found that management innovation has a positive effect on organizational efficiency and performance, and the improvement of organizational efficiency can greatly improve organizational performance. Modern healthcare management and practice strongly rely on data and scientific evidence (1). This requires the hospital management to be scientific and refined. Therefore, how to take management innovation as a starting point for fine management has become a question that many managers and scholars think about. Based on the traditional Activity-Based Costing (ABC), Professor Kaplan and Steven Anderson (2004) proposed the Time-Driven Activity Based Costing (TDABC), which greatly overcomes the shortcomings of the original method in the implementation process, such as expensive cost, time-wasting, low accuracy, etc. It integrates the differences of different business types into the time equation, and automatically allocates the resource consumption to the cost object by using the equation (2). This innovative management method also brings the dawn for hospital managers to improve the operational efficiency and performance of hospitals. At present, many scholars have applied TDABC to hospitals. Han Lin and other scholars believe that the indirect cost accounting method of Nursing Service based on time-driven activity-based costing can more accurately allocate the total indirect cost of nursing service to each nursing service item (3). Moreover, Zhang Bo-ping and other scholars found that TDABC can simplify the cost accounting procedures of obstetric medical services, improve the accuracy of accounting as well as provide data support for hospital project pricing (4). Some scholars, combined with the practices of the hospital, use time-driven activity-based costing (ABC) to calculate the cost of different treatment plans, to help hospital managers to determine the value of each scheme (5). Other researchers have compared traditional methods with TDABC and found that while TDABC systems require more data to implement, they still support that the TDABC model is superior to the traditional one (6). Furthermore, according to the characteristics of TDABC accounting process, Chen Shu and other scholars carried on the cost accounting of 9 medical service items in ultrasound department of Sample Hospital and discussed the establishment of hospital cost management and accounting system, which can make the cost accounting of hospital items convenient and accurate (7). Some scholars have proposed the fuzzy logic-TDABC model (FL-TDABC) to estimate the cost of medical services more accurately under uncertain conditions, in addition, it can also help hospital managers to make appropriate decisions on service capacity, capital budget, cost control and so on (8). In general, based on a comprehensive and detailed review of past research, some scholars also point out that TDABC is a value-based approach, which can not only save cost, but also drive to make the transition from fee-for service to value-based systems, thus provides a clearer concept of cost (9).

According to the study of domestic and foreign literature, the traditional ABC is difficult to implement due to its high application cost, while the TDABC is simpler, more economical, and more efficient. However, the current literatures only focus on accounting and does not involve a complete cost management system. In view of the above, this paper takes the cost management of public hospitals as the object, introduces the TDABC into this field, and provides reference for the effective implementation of cost management in public hospitals.

## Construction of the TDABM System for Grade-A Tertiary Hospitals

### Description of the TDABM System of Grade-A Tertiary Hospitals

The new “Hospital Accounting System” requires hospitals to establish a cost management system. According to the new medical reform, this paper divides cost management into cost budgeting, cost accounting, cost control, and cost performance in accordance with the process of hospital cost management. Therefore, the TDABM system in this paper is also consist of 4 components: time-driven cost budget, which operates as determination for performance targets and determination for the accuracy requirements for cost accounting; time-driven cost accounting, the accuracy of which directly influences the cost prediction, control and performance in the hospital; time-driven cost control, which operates as analysis of cost difference causes and original cost standard adjustments; time-driven cost performance, which operates as a new data basis for cost forecasting in the following stage.

**Figure 1:**
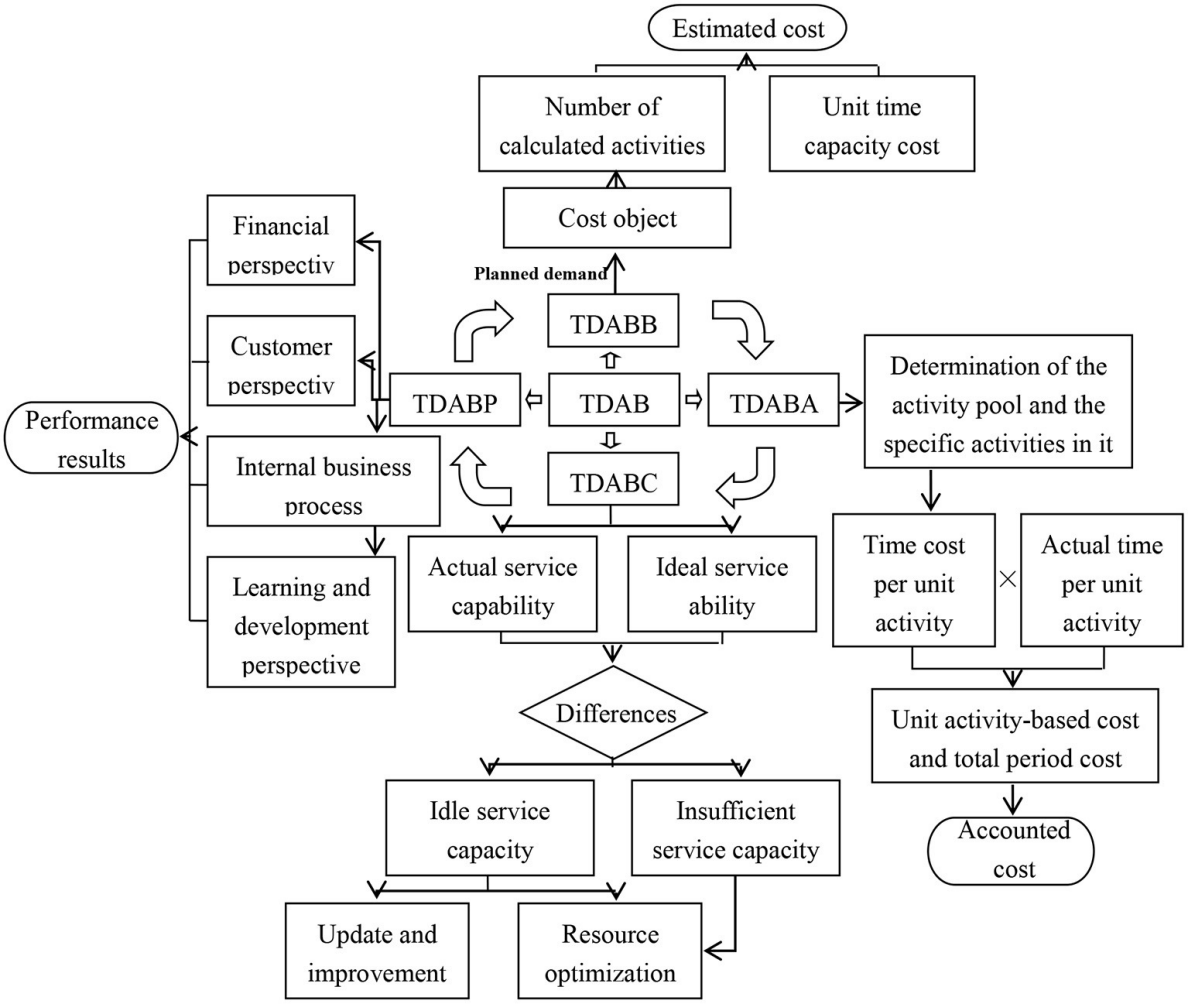
The TDABM system of Grade-A tertiary hospitals.

These four components form a closed cost management closed system, and each department provides cost management information for the next department. Specifically, the TDABM system of public hospitals in China is shown in Figure 1.

### The Time-Driven Activity-Based Cost Budget

The time-driven activity-based cost budget uses the energy consumed per unit of time to calculate costs such as services, products, and estimated consumed resources, to predict the structure and quantity of products, orders, services, customers, and to predict required resources based on unit energy costs.

For the activity-based cost budget of the hospitals, the calculation steps are as follows:

(1) Determine the activities required for various medical services based on the known demand for medical services. (2) Forecast the service demand for the next period. (3) Determine the number of activities consumed by the unit service and multiply it by the predicted demand of the service to predict the amount of work in the next business period. (4) Calculate the working time required for the next period by multiplying the activity demand by the unit work time. (5) Determine the energy consumption cost per unit time. In the forecast, the unit time energy consumption cost can refer to the enterprise history or the industry average unit time capacity cost. (7) Sum up all the operating costs of providing a certain medical service to obtain the estimated total cost. (8) Fill in the budget form and form a budget target.

### The Time-Driven Activity Cost Accounting

Hospital cost accounting is the cost accumulation and distribution of all the hospital business activities. The total cost and unit cost are calculated according to the object of accounting treatment.

The TDABC-based hospital costing process includes the following steps:

(1) Determine the accounting department and accounting items. Analyze departmental income, determine relevant medical service items, and work out the collection period of project accounting data. Generally speaking, the data collection period is 1 year. (2) Collect the income, cost, and medical project data of the accounting department. (3) Determine the activity. At this step, the cost information activity needs to be determined. For the hospital, the operation refers to the various processes or links in the medical service process, such as diagnosis, treatment, surgery, and so on. (4) In order to simplify the model, a certain degree of integration is required. When performing activity consolidation, it is best to have the same people performing the process to facilitate the cost allocation and calculation. (5) Determine the production cost rate. The capacity cost rate can be calculated by Equation 1:


(1) $$\begin{array}{r} \text{The capacity cost rate}=\frac{\text{Capital required by the department}}{\text{Energy required for actual capacity}} \end{array}$$

Capacity cost refers to the cost of the resources used to perform the operations, typically including the wages of the employee, equipment, technology costs, the cost of the rental office, and any other expenses. Actual capacity is measured in terms of unit time; it refers to the actual capacity of the resources, not the theoretical maximum capacity. Kaplan and Anderson^错误!未找到引用源^ suggested that the actual capacity used is 80 to 85% of the maximum theoretical capacity of the staff, which considers the non-production time spent on employee work. (6) Estimate working time. At this stage, managers estimate the time it takes to complete a single job. A unit's work means the time an employee completes a single order. (7) Determine the time equation. Usually, the different characteristics of the business determine the length of time it takes. The TDABC uses an additive linear equation to reflect the resource capacity requirements of different types of activities. From a mathematical point of view, the time equation can be written as Equation 2:


(2) $$\begin{array}{r} \text{Time required for the process}\\ =\text{Sum of time required for each activity}\\ =\left({\text{α}}_{0}+{\text{α}}_{1}{\text{X}}_{1}+{\text{α}}_{2}{\text{X}}_{2}+{\text{α}}_{3}{\text{X}}_{3}+\ldots +{\text{α}}_{\text{i}}{\text{X}}_{\text{i}}\right) \end{array}$$

In Equation 2, α_0_ is the standard time of basic work (for example, 10 min); α_*i*_ is the time required for the extra i-th activity (for example, 2 min); and *X*_*i*_ is the number of additional activities (for example, the number of projects). (8) Determine the cost driver rate. In the TDABC, the cost driver rate of an activity is determined by the capacity cost rate and the estimated time of an activity unit. For example, if the capacity cost rate for an assignment is 2 yuan/min, it is estimated that the assignment takes 15 min to complete, and the cost driver for completing the assignment is 30 yuan. (9) Allocate the activity costs to the cost target. The cost is allocated to the cost target by multiplying the cost driver rate by the actual number of cost driver units.

### The Time-Driven Activity Cost Control

In the TDABC, time is the most basic resources and the most important basis for measuring the overall operational efficiency of a system. There are usually four cases of time consumption: service time, inspection time, transportation time, and invalid time. The longer the task cycle of a medical activity, the more cost it will generate. Therefore, by comparing the planned time with the actual execution time, the specific increment and its efficiency can be fully gasped. By increasing the efficiency value, eliminating or reducing non-value-added time, the cost is reduced accordingly.

In general, the following methods can be adopted to control the different nature of each job: (1) Activity maintenance. It is necessary to continue to maintain the necessary value-added operations. For example, the patient's consultation time, it is necessary to continue to maintain this necessary time. (2) Activity optimization. For non-essential value-added operations, integrate the same or similar tasks to minimize the resources and time spent on multiple activities, improve the efficiency of the operation, and simplify the operation process. For instance, the integrated utilization of idle equipment, the sale of long-unused equipment and so on. Equipment that can be used interchangeably between departments can be adjusted as much as possible to reduce the possibility of equipment being idle. (3) Activity improvement. Activity improvement is required for the necessary non-value-added activities and inefficient value-added activities, such as improving work efficiency, reducing the number of operations, and reducing work time, etc. For example, the long queue waiting time in the registration can be improved by adding a temporary window. (4) Activity elimination. Eliminate non-essential and non-value-added activities. Before the process optimization, eliminate the non-value-added content and operation links, according to the analysis results of the workflow. For the idle staff of the department, measures such as staff reduction should be considered. Unnecessary examinations of patients are also unnecessary and non-value-added activities and should be eliminated.

### The Time-Driven Activity Cost Performance

**Figure 2:**
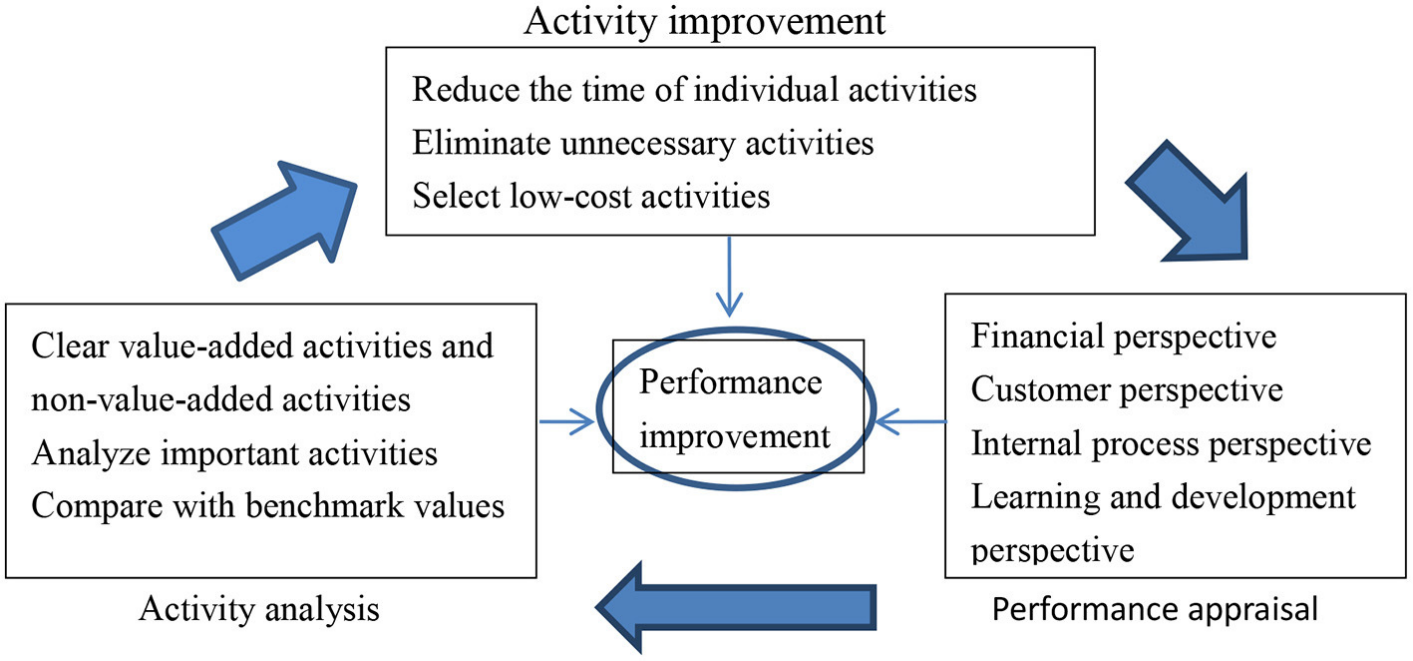
The conceptual framework of time-driven cost performance.

The time-driven activity cost performance is based on time-driven cost control. The time-driven cost control completes the steps of activity analysis and improvement, reduces the time of individual operations, eliminates unnecessary operations, selects low-cost operations, and uses the data after the activity improvement to do performance appraisal, and then improves performance. At the same time, activity analysis and improvement are themselves a means of performance improvement and an essential process (Figure 2).

**Figure 3:**
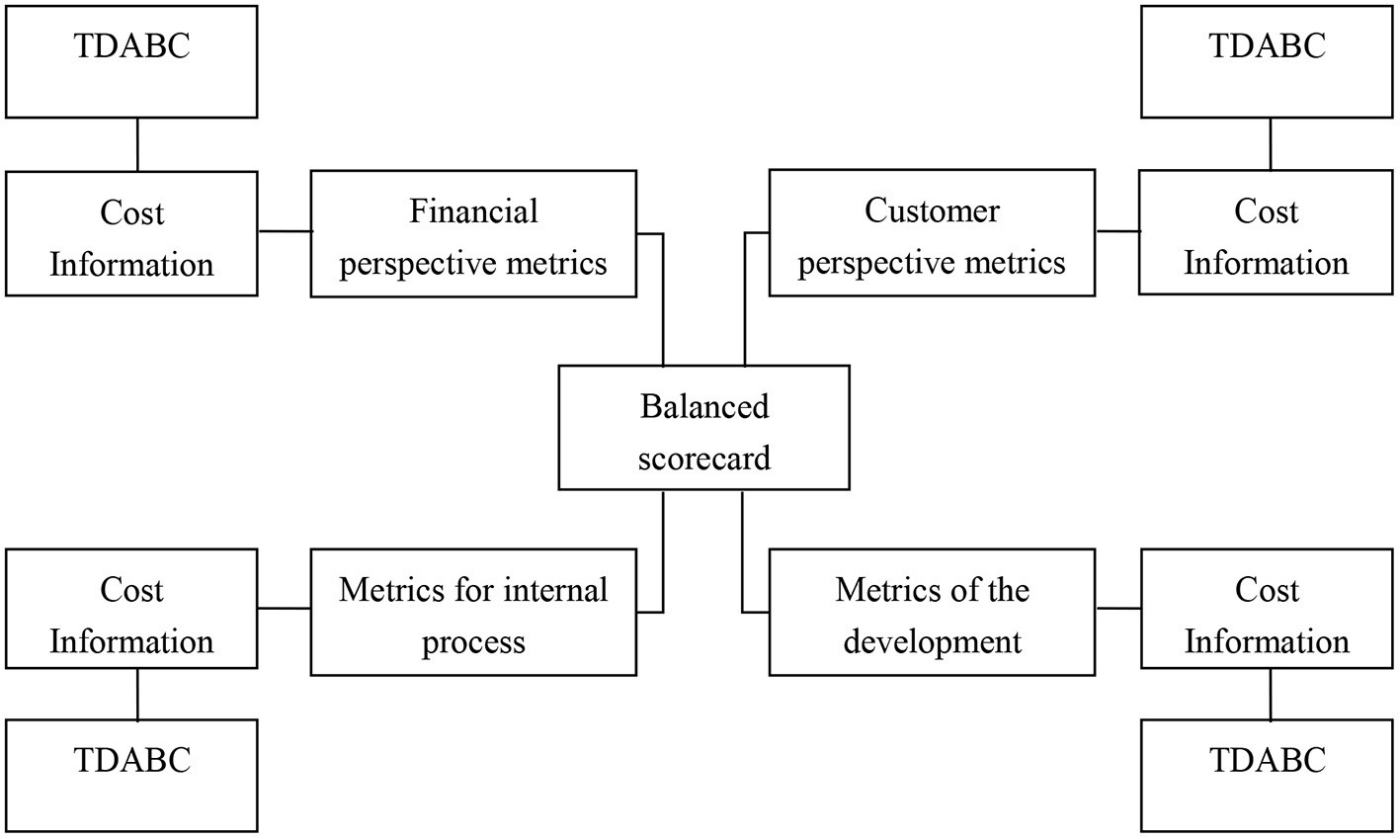
Combining balanced scorecard with TDABC.

Time-driven activity cost performance can be combined with the balanced scorecard. Applying the balanced scorecard to the cost assessment and the evaluation system can significantly improve the satisfaction of medical staff, not only because of economic returns, but also because of the opportunities to improve internal processes and community relationships, getting growth and learning. The Balanced Scorecard increases the concern about hospital intangible asset management, the knowledge value of medical staff and the relationship between doctors and patients, and increases patient participation, and makes the concept of cost performance run through every process of hospital operation, which is the driving tool to enhance the competitive advantage of hospitals (Figure 3).

## Research on the Application of Time-Driven Activity-Based Management: Evidence From JSPH Hospital

This section describes the organizational structure of the case hospital (JSPH Hospital), analyses the current problems in the cost management of this hospital and applies the constructed time-driven activity-based management to this hospital.

### Current Situation and Problems of Cost Management in JSPH Hospital

The establishment of cost management in JSPH Hospital is based on accounting department, finance department, supply department, information center, clothing center, supply center, pharmacy, medical library, and logistics service center for library materials. The cost management of this hospital is relatively well-established, and the computer management of the cost management department is working properly, providing technical support for the cost management work at JSPH Hospital.

However, there are still some problems in the cost management in JSPH Hospital, which are shown as follows.

(1) The hospital lacks human resources for cost accounting and the lack of a complete cost management system leads to the superficiality of cost budget, control, and performance, which makes JSPH Hospital under great pressure for survival.
(2) Cost accounting elements are not complete. Emphasis of cost management is placed on accounting for clinical and medical departments, while underestimating the costs of administrative and logistics services; The emphasis of accounting is on the direct costs of departments, rather than including all elements of indirect costs in the accounting. Therefore, the economic efficiency assessments and prediction based on this information loses its benchmark.
(3) Cost allocation is unreasonable. The current cost allocation practice of JSPH Hospital is directly proportional to the personnel in the Pharmacy Department, which is too simple and does not reflect the real cost of cost objects.

For these reasons, if JSPH Hospital continues to use the previous cost management methods and fails to pay attention to and solve the cost management problems, it will significantly restrict the development of the hospital, and the medical reform will also face great challenge. Therefore, it is especially necessary for JSPH Hospital to set up a perfect modern cost management framework, which should be constructed by time-driven activity-based costing, because the implementation and application of time-driven activity-based costing is the latest optimization of the existing cost management framework.

### Management Framework for JSPH Hospital

The first step is selecting a target segment. The target departments are selected according to the following criteria: accurate cost data can be obtained; daily operational processes are simple and fixed.

For these reasons, we chose the traditional Chinese medicine clinic of JSPH Hospital for the case study. According to the opinions of hospital experts, the business process of traditional Chinese medicine department is divided into three stages, which are namely triage, diagnosis, and comprehensive treatment. According to this process, the TCM department operations can be divided into 13 operational items: appointment with the renowned specialist, appointment with the chief physician, appointment with the deputy chief physician, appointment with the attending physician, acupuncture point therapy, subcutaneous injection, intramuscular injection, intravenous infusion, outpatient routine infusion observation fee, immersion therapy, external application of cream treating at acupuncture points in the way of “winter diseases treated in summer,” rapid quantification of dry chemical blood glucose.

#### JSPH Hospital Cost Budget

1) The average time needed for basic medical services in TCM departments is shown in Table 1.
2) According to past experience, the estimated production cost rate is 0.8 yuan per minute. Thus, Table 2 can be estimated as follows.

**Table 1:** Time consumption of medical operation unit.

**Workflow**	**Unit time taken (min)**
Appointment with the renowned specialist	25
Appointment with the chief physician	20
Appointment with the deputy chief physician	18
Appointment with the attending physician	15
Acupuncture point therapy	3
Subcutaneous injection	10
Intramuscular injection	5
Intravenous infusion	20
Outpatient routine infusion observation fee	25
Immersion therapy	30
Treating asthma in the way of “winter diseases treated in summer”	3
Rapid quantification of dry chemical blood glucose	5

**Table 2:** Costs budget for traditional Chinese medicine departments.

**Workflow**	**Unit time taken** **(min)**	**Expected cost driver Rate** **(yuan/case)**	**Last cost driver quantity** **(case)**	**Projected cost driver volume for the month** **(case)**	**Estimated burden sharing** **(yuan)**
Appointment with the renowned specialist	25	20.00	1,254	1504.80	30096.00
Appointment with the chief physician	20	16.00	198	237.60	3801.60
Appointment with the deputy chief physician	18	14.40	1,386	1663.20	23950.00
Appointment with the attending physician	15	12.00	1,986	2383.20	28598.00
Acupuncture point therapy	3	2.40	2,984	3580.80	8593.90
Subcutaneous injection	10	8.00	29	34.80	278.40
Intramuscular injection	5	4.00	98	117.60	470.40
Intravenous infusion	20	16.00	5	6.00	96.00
Outpatient routine infusion observation	25	20.00	9	10.80	216.00
Immersion therapy	30	24.00	234	280.80	6739.20
Treating asthma in the way of “winter diseases treated in summer”	3	2.40	199	238.80	573.12
Rapid quantification of dry chemical blood glucose	5	4.00	6	7.20	28.80
Total					103442.00

The results of the budget can be used as the basis for the next cost budget, and as a reference for subsequent cost control and cost performance.

#### Cost Accounting of JSPH Hospital

1) the total cost of traditional Chinese medicine department for 1 month is shown in Table 3.
  The average working time of TCM department staff is 8 h per day, and the average person works 22 days per month. Then the theoretical service capacity of the department can be calculated as follows:
  T = 19 × 8 × 22 × 60 = 200,640 (min).
  The actual service capacity was 160,512 min (200,640 × 80%).
  The service cost rate was yuan 0.81 per min (130266.64/160,512).
2) Determine the amount of cost drivers for each Workflow, as shown in Table 4.
3) Calculate the cost driver rate of each workflow, as shown in Table 5.
4) Calculate the final cost of the cost object, as shown in Table 6.

**Table 3:** Total cost of outpatient department of Traditional Chinese medicine.

**Category of expense**	**Cost (yuan)**
Wages	40323.52
Cost of sanitary materials	42815.92
Equipment related fees	13086.51
Property management fees	4303.34
Water bill	793.11
Electricity bill	4730.31
ADMINISTRATIVE expenses	824.09
Other expenses	11476.39
Allocation of management departments	6453.26
Allocation of medical auxiliary departments	5460.18
Total	130266.64

**Table 4:** Activity-based cost drivers of TCM departments.

**Workflow**	**Times**
Appointment with the renowned specialist	1,897
Appointment with the chief physician	219
Appointment with the deputy chief physician	2,278
Appointment with the attending physician	3,198
Acupuncture point therapy	2,468
Subcutaneous injection	24
Intramuscular injection	56
Intravenous infusion	12
Outpatient routine infusion observation fee	12
Immersion therapy	233
Treating asthma in the way of “winter diseases treated in summer”	207
Rapid quantification of dry chemical blood glucose	9

**Table 5:** Cost driver rate of each activity in TCM departments.

**Workflow**	**Unit capacity consumption** **(min)**	**Unit capacity cost** **(yuan/min)**	**Cost driver rate** **(yuan/case)**
Appointment with the renowned specialist	25	0.81	20.25
Appointment with the chief physician	20	0.81	16.20
Appointment with the deputy chief physician	18	0.81	14.58
Appointment with the attending physician	15	0.81	12.15
Acupuncture point therapy	3	0.81	2.43
Subcutaneous injection	10	0.81	8.10
Intramuscular injection	5	0.81	4.05
Intravenous infusion	20	0.81	16.20
Outpatient routine infusion observation fee	25	0.81	20.25
Immersion therapy	30	0.81	24.30
Treating asthma in the way of “winter diseases treated in summer”	3	0.81	2.43
Rapid quantification of dry chemical blood glucose	5	0.81	4.05

**Table 6:** Final cost of each operation in TCM departments.

**Workflow**	**Material unit price** **(yuan)**	**Unit capacity consumption (min)**	**Cost driver rate (yuan/case)**	**Cost driver quantity (case)**	**Indirect cost allocation (yuan)**	**Direct cost (yuan)**	**Total cost (yuan)**
Appointment with the renowned specialist	0.50	25	20.25	1,897	38414.25	948.50	39363.00
Appointment with the chief physician	0.50	20	16.20	219	3547.80	109.50	3657.30
Appointment with the deputy chief physician	0.50	18	14.58	2,278	33213.24	1139.00	34352.00
Appointment with the attending physician	0.50	15	12.15	3,198	38855.70	1599.00	40455.00
Acupuncture point therapy	7.00	3	2.43	2,468	5997.24	17276.00	23273.00
Subcutaneous injection	5.00	10	8.10	24	194.40	120.00	314.40
Intramuscular injection	5.00	5	4.05	56	226.80	280.00	506.80
Intravenous infusion	2.00	20	16.20	12	194.40	24.00	218.40
Outpatient routine infusion observation fee	0.50	25	20.25	12	243.00	6.00	249.00
Immersion therapy	30.00	30	24.30	233	5661.90	6990.00	12652.00
Treating asthma in the way of “winter diseases treated in summer”	30.00	3	2.43	207	503.01	6210.00	6713.00
Rapid quantification of dry chemical blood glucose	8.00	5	4.05	9	36.45	72.00	108.45
Total cost (yuan)					127088.19	34774.00	150574.00

Time-driven cost accounting makes the cost accounting unit more detailed than the performance appraisal unit, which provides a more accurate assessment basis for hospital performance management.

#### JSPH Hospital Cost Control

As can be seen from Table 7, in this example, the necessary leisure time of the staff and the intermittent of patient payment (which cannot be continuous) have been fully considered when setting effective working hours. Therefore, the finally calculated unused working hours of 3,613 min (about 7 working days) can be used for other purposes, such as training medical personnel, so as to improve the working efficiency.

**Table 7:** Idle production capacity of each operation in the B department.

**Workflow**	**Unit capacity consumption** **(min)**	**Cost driver quantity** **(case)**	**Total time** **(min)**	**Total cost** **(yuan)**
Appointment with the renowned specialist	25	1,897	47,425	39362.75
Appointment with the chief physician	20	219	4,380	3657.30
Appointment with the deputy chief physician	18	2,278	41,004	34352.24
Appointment with the attending physician	15	3,198	47,970	40454.70
Acupuncture point therapy	3	2,468	7,404	23273.24
Subcutaneous injection	10	24	240	314.40
Intramuscular injection	5	56	280	506.80
Intravenous infusion	20	12	240	218.40
Outpatient routine infusion observation fee	25	12	300	249.00
Immersion therapy	30	233	6,990	12651.9
Treating asthma in the way of “winter diseases treated in summer”	3	207	621	6713.01
Rapid quantification of dry chemical blood glucose	5	9	45	108.45
Total actual hours spent			156,899	150574.19
Total effective hours			160,512	130266.64
Total unused hours			3,613	−20307.55

#### JSPH Hospital Cost Performance

JSPH Hospital has found measures that can be used for reference from the methods of enterprise cost management and has established a cost-centered system. Taking traditional Chinese medicine as an example, its performance plan is set as shown in Table 8.

**Table 8:** Formulation of B departmental performance indicators.

**Perspective**	**Secondary index**	**Level three index**	**Evaluation criterion**
Financial perspective (30%)	Economic efficiency (60%)	Rate of return (50%)	Actual value/target value × comprehensive weight
		Material consumption rate per 100 yuan of income (50%)	(2- actual value/target value) ^*^ comprehensive weight
		Per capita outpatient fee (30%)	According to the interval standard
		Per capita hospitalization expenses (30%)	According to the interval standard
		Pharmaceutical revenue to business revenue ratio (40%)	A proportionate reduction or increase above or below a standard
Patient perspective (30%)	Patient trust (60%)	Patient satisfaction rate (20%)	Actual value/target value × comprehensive weight
		Outpatient growth (40%)	According to the interval standard
		Increase in hospitalized patients (40%)	According to the interval standard
	Zero defect management (40%)	Number of patient complaints (40%)	Deduct 1 point for each case of increase
		Medical compensation (60%)	Deduct 1 point for each complaint
Internal processes (25%)	Service efficiency (30%)	Survival rate of patients after rescue (30%)	Deduct 1 point for every 1% reduction
		Outpatient visits (30%)	Actual value/target value × comprehensive weight
		Average length of staying in hospital (40%)	Deduct 1 point for each additional day
	Service quality (70%)	Comprehensive hospitalization index (30%)	Actual value/target value × comprehensive weight
		Clinical pathway utilization rate (10%)	Deduct 1 point for every 1% reduction
		Diagnostic coincidence rate (10%)	Deduct 1 point for every 1% reduction
		Grade A rate of hospitalized medical records (10%)	Deduct 1 point for every 1% reduction
		Cure rate of patients (10%)	Deduct 1 point for every 1% reduction
		In-patients' satisfaction with nursing care (10%)	According to the interval standard
		Quality of nursing (20%)	Actual value/target value × comprehensive weight
Learning growth (15%)	R&D (40%)	Research project award (50%)	Actual value/target value × comprehensive weight
		Paper publishing (50%)	Actual value/target value × comprehensive weight
	Staff growth (60%)	Percentage of teaching staff (30%)	Actual value/target value × comprehensive weight
		Participation in continuing education (30%)	Actual value/target value × comprehensive weight
		Talent pool (25%)	Actual value/target value × comprehensive weight
		Daily process management (15%)	Actual value/target value × comprehensive weight

The target performance value is set at the beginning of the year according to the monthly average performance completion of the previous year, and the actual value is subject to the actual situation of the department in this month. The specific performance is shown in Table 9.

**Table 9:** Performance evaluation of each department.

**Indicators for performance assessment**	**Department of traditional Chinese medicine**	
	**Target value**	**Actual value**
Rate of return (50%)	11.99	11.91
Material consumption rate per 100 yuan of income (50%)	41.86	39.63
Per capita outpatient fee (30%)	307.68	209.38
Per capita hospitalization expenses (30%)	41820.40	35057.60
Pharmaceutical revenue to business revenue ratio (40%)	100.00	96.60
Patient satisfaction rate (20%)	25.67	22.39
Outpatient growth (40%)	10.00	9.16
Increase in hospitalized patients (40%)	20.00	38.97
Number of patient complaints (40%)	0	0
Medical compensation (60%)	0	0
Survival rate of patients after rescue (30%)	14.51	12.84
Outpatient visits (30%)	85.00	100.00
Average length of staying in hospital (40%)	7607.00	7542.00
Comprehensive hospitalization index (30%)	90.00	90.50
Clinical pathway utilization rate (10%)	95.00	98.99
Diagnostic coincidence rate (10%)	100.00	96.30
Grade A rate of hospitalized medical records (10%)	95.00	95.30
Cure rate of patients (10%)	80.00	96.00
In-patient's satisfaction with nursing care (10%)	100.00	99.59
Quality of nursing (20%)	70.00	50.00
Research project award (50%)	100.00	100.00
Paper publishing (50%)	100.00	100.00
Percentage of teaching staff (30%)	100.00	100.00
Participation in continuing education (30%)	100.00	75.00
Talent pool (25%)	22.5	20.60
Daily process management (15%)	13.50	14.40

Through the results of performance appraisal of clinical departments (as shown in Table 10), the realization of the objectives can be obtained, the factors that have an impact on performance can be analyzed, and effective measures can be taken to improve these factors.

**Table 10:** Comprehensive scores of performance assessment of clinical departments.

**Perspective**	**Secondary index**	**Level three index**	**Department of traditional Chinese medicine**
Financial perspective (30%)	Economic efficiency (60%)	Rate of return (50%)	89.42
		Material consumption rate per 100 yuan of income (50%)	90.00
		Per capita outpatient fee (30%)	48.00
		Per capita hospitalization expenses (30%)	32.24
		Pharmaceutical revenue to business revenue ratio (40%)	33.60
Patient perspective (30%)	Patient trust (60%)	Patient satisfaction rate (20%)	48.00
		Outpatient growth (40%)	34.78
		Increase in hospitalized patients (40%)	54.96
	Zero defect management (40%)	Number of patient complaints (40%)	72.00
		Medical compensation (60%)	48.00
Internal processes (25%)	Service efficiency (30%)	Survival rate of patients after rescue (30%)	72.00
		Outpatient visits (30%)	22.50
		Average length of staying in hospital (40%)	30.00
	Service quality (70%)	Comprehensive hospitalization index (30%)	22.50
		Clinical pathway utilization rate (10%)	17.50
		Diagnostic coincidence rate (10%)	17.50
		Grade A rate of hospitalized medical records (10%)	33.71
		Cure rate of patients (10%)	17.50
		In-patients' satisfaction with nursing care (10%)	17.30
		Quality of nursing (20%)	52.28
Learning growth (15%)	R&D (40%)	Research project award (50%)	0.00
		Paper publishing (50%)	30.00
	Staff growth (60%)	Percentage of teaching staff (30%)	30.00
		Participation in continuing education (30%)	27.00
		Talent pool (25%)	20.25
		Daily process management (15%)	20.60

It can be seen from Table 10 that the proportion of the cost to the income of the department has reached the ideal value, and the per capita outpatient expenses have also been relatively reduced, but the satisfaction of patients, the quality of care, the number of continuing education and the talent pool need to be improved. The department should further improve nursing quality and patient satisfaction and attach importance to personnel training and reserve.

Previously, the cost performance evaluation of JSPH Hospital was mainly based on financial indicators. At present, many new indicators, such as internal processes, have been added to the cost performance system of JSPH Hospital, and the evaluation scope has also increased. The cost of each operating unit should not only be compared with the past performance, but also with the performance of other operating units, with the average level of the same region and industry, and with the advanced benchmark of the same industry. It depends not only on the financial indicators such as workload, department income, balance of payments, but also on the satisfaction of patients and staff, and the standard level of internal management. JSPH Hospital should pay attention to immediate interests, but also the long-term interests. Only in this way can the hospital achieve more stable and sustainable development.

## Conclusions

The conclusions of this study are as follows: (1) TDABC provides a reliable indicator for the hospital cost budget. The time-driven activity-based cost budget can effectively serve patients and reduce medical costs. (2) TDABC provides accurate data for hospital cost accounting.

The complexity and uncertainty of medical procedures greatly increase the difficulty of hospital cost management. TDABC can use time equations to accurately calculate the time required for complex medical services and use the rich data resources of existing systems to calculate the cost of unit work. (3) TDABC provides a stable system for hospital cost control. When the time required by the unit changes or there is new work, the administrator only needs to adjust or add new data, and it does not affect the entire model structure. Therefore, TDABC can adapt well to changes in the internal and external operating environment of the hospital, effectively saving management costs for the hospital. (4) TDABC provides a scientific basis for hospital performance appraisal. Through TDABC, managers can discover the cost of wasted work hours that are not fully utilized by health care providers, provide valuable reference for hospital performance appraisal, and a scientific basis for improving process efficiency and cost savings. (5) TDABC provides a complete system for hospital cost management. TDABC can realize the cost management of the whole process and incorporate time factors into the cost management, which helps medical staff to save time and improve work efficiency, and further enhances hospital competitiveness.

## Data Availability Statement

The original contributions presented in the study are included in the article/supplementary material, further inquiries can be directed to the corresponding author/s.

## Author Contributions

QJ: formulate the general idea and carry on the practice inquiry as well as write the part first draft. XZ: wrote the first draft and visualize result. LC: review and revision of the first draft. ZZ: on the spot investigation and data analysis in hospital. YC: collect relevant data and revise article and check. All authors contributed to the article and approved the submitted version.

## Funding

This work was funded by grants of committee project research on budget management of Public Hospital of National Social Science Fund of China (No. 21FGLB010) and committee project research on dynamic stability of supply chain cost allocation in heterogeneous environment of National Natural Science Foundation of China (No. 71772036).

## Conflict of Interest

The authors declare that the research was conducted in the absence of any commercial or financial relationships that could be construed as a potential conflict of interest.

## Publisher's Note

All claims expressed in this article are solely those of the authors and do not necessarily represent those of their affiliated organizations, or those of the publisher, the editors and the reviewers. Any product that may be evaluated in this article, or claim that may be made by its manufacturer, is not guaranteed or endorsed by the publisher.
